# Placental damage comparison between preeclampsia with COVID-19, COVID-19, and preeclampsia: analysis of caspase-3, caspase-1, and tumor necrosis factor-alpha expression

**DOI:** 10.1016/j.xagr.2023.100234

**Published:** 2023-06-10

**Authors:** Muhammad Adrianes Bachnas, Aira Oklatihana Putri, Elita Rahmi, Rosita Alifa Pranabakti, Nutria Widya Purna Anggraini, Lini Astetri, Eric Edwin Yuliantara, Wisnu Prabowo, Supriyadi Hari Respati

**Affiliations:** 1 Faculty of Medicine, Division of Maternal-Fetal Medicine, Department of Obstetrics and Gynecology, Dr Moewardi General Hospital, Sebelas Maret University, Solo, Indonesia (Drs Bachnas, Aggraini, Astetri, Yuliantara, and Prabowo); 2 Faculty of Medicine, Department of Obstetrics and Gynecology, Dr Moewardi General Hospital, Sebelas Maret University, Solo, Indonesia (Drs Putri, Rahmi, and Pranabakti); 3 Faculty of Medicine, Division of Obstetrics, Community, and Social Science, Department of Obstetrics and Gynecology, Dr Moewardi General Hospital, Sebelas Maret University, Solo, Indonesia (Dr Respati)

**Keywords:** COVID-19, placental damage, preeclampsia

## Abstract

**BACKGROUND:**

Some studies have reported that preeclampsia with COVID-19 significantly increases the risk of adverse perinatal outcomes to nearly 3-fold in normal pregnancy. In theory, the pathophysiology of preeclampsia increases perinatal mortality and morbidity starting from placental injury, which is also believed to share a common pathway with COVID-19. Moreover, major placental injuries could be apoptotic, necrotic, or pyroptotic.

**OBJECTIVE:**

This study aimed to compare the placental damage between Preeclampsia with COVID-19, COVID-19, and preeclampsia in apoptotic, necrotic, or pyroptotic injuries.

**STUDY DESIGN:**

This was an observational analytical study with a cross-sectional setting. A total of 72 pregnant women were admitted to the hospital with diagnoses of preeclampsia with COVID-19, preeclampsia, and COVID-19. The diagnosis for preeclampsia was following the International Federation of Gynecology and Obstetrics criteria with at least 1 of the severe features. Patients with COVID-19 were eligible if they had a confirmatory polymerase chain reaction test with moderate to severe clinical degree. The placentas were taken after delivery, and the parameters were quantified with immunohistochemistry tests for caspase-3, caspase-1, and tumor necrosis factor-alpha representing apoptotic, pyroptotic, and necrotic pathways, respectively.

**RESULTS:**

Pregnancies complicated by both COVID-19 and preeclampsia, preeclampsia, and COVID-19 significantly had the highest placental damage on apoptotic, pyroptotic, and necrotic pathways shown from caspase-3, caspase-1, and tumor necrosis factor-alpha expressions in the placenta (*P*<.05). Moderate to severe degree of COVID-19 resulted in higher placental damage than preeclampsia in all 3 forms (*P*<.05). The apoptotic process was the most prominent among the pathways.

**CONCLUSION:**

Preeclampsia with COVID-19 showed significant placental damage, with major changes related to apoptosis, inflammation, and necrosis. Our data support poor perinatal outcomes of pregnancies complicated by both preeclampsia and COVID-19.

**AJOG Global Reports at a Glance**

**Why was this study conducted?**

A comparison of placental damage between Preeclampsia with COVID-19, COVID-19, and preeclampsia has never been studied or reported, explaining the poor perinatal outcomes in pregnancies complicated by both COVID-19 and preeclampsia.

**Key findings**

A very high expression of caspase-3 in the placenta of pregnant patients with both COVID-19 and preeclampsia showed a major apoptotic process, which is related to the extreme deterioration of function that results in an increased number of perinatal death, including intrauterine fetal death and other perinatal morbidities. In addition, the inflammation and necrotic processes were prominently shown by overexpression of caspase-1 and tumor necrosis factor-alpha (TNF-α), which were significantly high in pregnancies complicated by COVID-19 and pregnancies complicated by preeclampsia.

**What does this add to what is known?**

Our results have given scientific reasoning of the pathophysiological process of placental damage that causes poor perinatal outcomes in Preeclampsia with COVID-19 through the measurements of caspase-1, caspase-3, and TNF-α expressions in the placenta. This has never been studied or published and has given a thoughtful idea for a management solution.

## Introduction

The connection between COVID-19 and adverse pregnancy outcomes has been generally reported.^1^ In addition, its relationship with preeclampsia has been investigated.^2^^,^^3^ Some data showed that women with a history of a positive risk screening of preeclampsia in the first trimester of pregnancy have a significantly high risk of developing COVID-19.^4^ Moreover, data explained that COVID-19 during pregnancy is strongly associated with preeclampsia, which can be assumed because of placental origin.^5^ Both conditions that occur at the same time would be associated with the worst maternal and perinatal outcomes.^5^

However, there is no study that reported the exact pathomechanism in the placenta. Some studies are related to viremia and viral invasion in the placenta with placentitis, cytokine storm that affects the placental cells, blood clot that results in infarction and ischemia, and accelerated cell death program. However, all those studies were not supported with robust data; moreover, some studies reported no significant change at all.^6^^,^^7^ In 2017, a systematic review and meta-analysis of preeclampsia concluded that the abnormality of the placenta in women with preeclampsia is 7-fold higher than in women with normal pregnancy, but the lesions were not specific.^8^ A published well-design study stated that the major changes in a placenta of a patient with preeclampsia were ischemia, inflammation, and cell death.^9^

In apoptosis, caspase-3 plays a crucial role in mitochondrial events. Caspase-3 overactivity has been shown to trigger loss of mitochondrial function that leads to apoptosis. Inhibition of this mechanism leads to significant prevention of cell death.^10^ In inflammation, caspase-1 is believed to have a pivotal role in the massive production of inflammatory agents and cytokine release.^11^ In preeclampsia, inflammation mainly occurs in the placenta and in maternal vascular injury.^12^ Placental inflammatory disorders will lead to perinatal morbidity and mortality.^13^ Although the level of inflammation is relatively low, it is chronic and occurs long enough to cause systemic endothelial vascular injury.^13^ However, COVID-19 has a different story. It has an acute and massive release of cytokines, often called a cytokine storm. Because of its massive size and the firmness of the inflammation, it often results in massive thrombosis, followed by multiorgan failure.^12^^,^^14^ In the same conjunction point between COVID-19 and the preeclampsia pathways, the vascular damage, inflammation, and thrombosis lead to ischemia and infarction, which will be followed by increasing synthesis of tumor necrosis factor-alpha (TNF-alpha) related to necrosis.^15^, ^16^, ^17^

This study aimed to investigate the roles of caspase-1, caspase-3, and TNF-alpha expressions in the placenta of pregnant women with both preeclampsia and COVID-19, COVID-19, and preeclampsia and to determine whether preeclampsia with COVID-19 damages the placenta.

## Materials and Methods

This study was an observational study with a cross-sectional design and was approved by the ethics board of Dr Moewardi General Hospital and Sebelas Maret University. The study participants were pregnant women who were admitted and delivered at Dr Moewardi General Hospital, a tertiary academic hospital of Sebelas Maret University in Solo, Indonesia. We enrolled 72 pregnant women from March 2021 to September 2022 as they met the inclusion and exclusion criteria and were willing to participate. Preeclampsia was categorized using the American College of Obstetricians and Gynecologists criteria, and COVID-19 was categorized using the World Health Organization and Centers for Disease Control and Prevention criteria and by considering the earliest publication related to COVID-19 in pregnancy.^18^, ^19^, ^20^ The participants were grouped into 3 categories: Preeclampsia with COVID-19, COVID-19 only, and preeclampsia only. We included only moderate to severe grade of COVID-19 and severe preeclampsia. Major confounding factors, such as gestational age at delivery, birthweight, diabetes mellitus, chronic infection, maternal age, and parity, were considered. In addition, maternal and fetal clinical outcomes were reported.

The placenta was handled right after delivery and stained using specific immunohistochemistry assays to assess caspase-3, caspase-1, and TNF-alpha expression levels. The quantification of expression levels was performed using the Remmele scoring system or the immunoreactive score (IRS). A microscopic view was first performed at 100 times magnification and finished at 400 times magnification to visualize the positive cell percentage and intensity of the stain representing the histopathologic condition of the placenta. The higher the intensity and the more cells are involved after the immunohistochemistry staining, the more it represents the apoptotic process in the placenta by caspase-3, the inflammation process by caspase-1, and the necrosis process by TNF-alpha. The IRS comes from the multiplication of the positive cell percentage (0–4) and the intensity of the reaction (0–3). The maximum IRS is 12, and the minimum IRS is 0.^21^, ^22^, ^23^ The visualization was focused on the syncytiotrophoblast cells, the outermost layer of the human placenta, which represents the most important site in the placenta for nutrient and oxygen transportation and for drug, metabolite, and waste product exchange between the maternal and fetal circulations (Figure). Immunohistochemistry assay results were collected from all placentas of the 3 groups—preeclampsia only, COVID-19 only, and Preeclampsia with COVID-19—and compared in statistical analysis as the objective of the study, which is the placental damage from 3 major pathways, apoptosis, inflammation, and necrosis.

**Figure:**
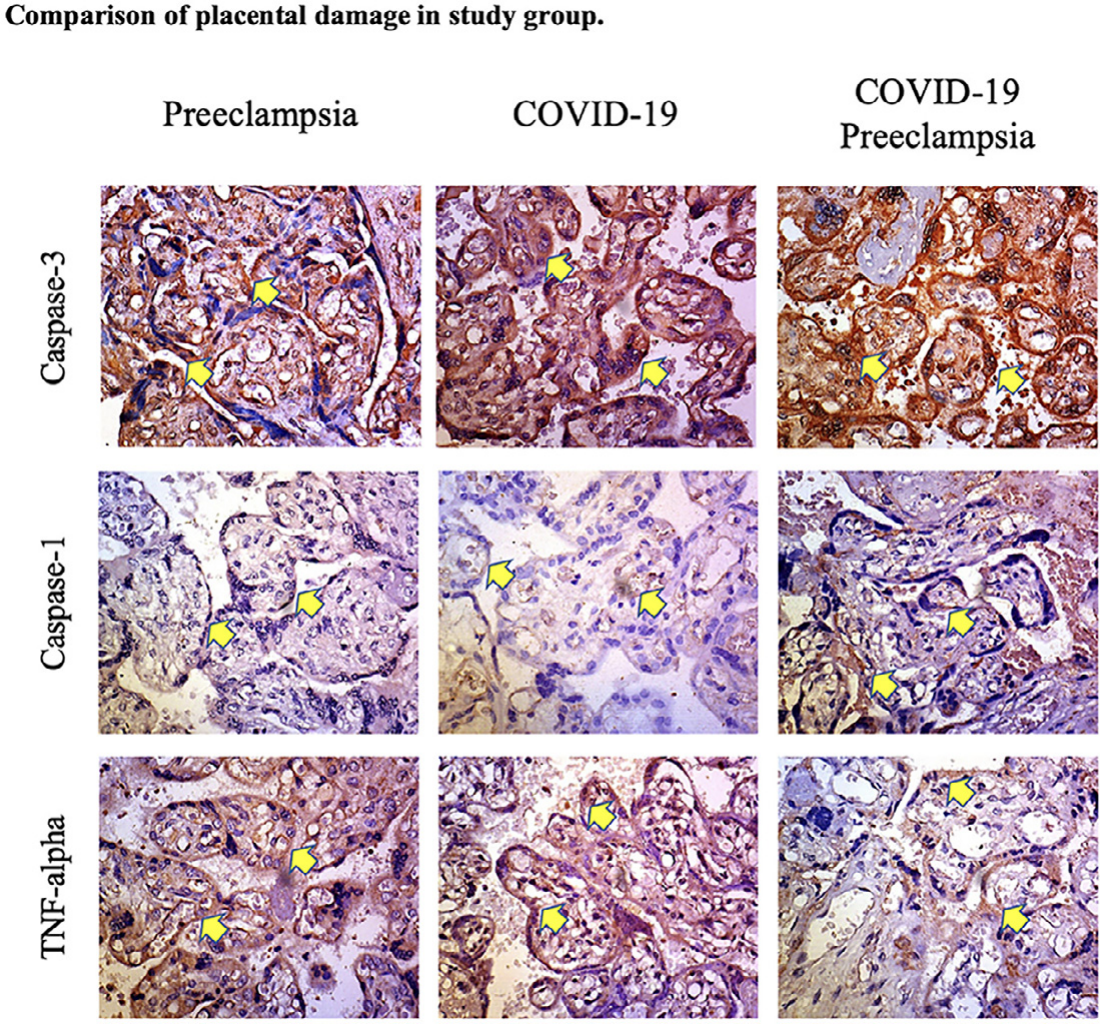
Comparison of placental damage in study group The images are microscopic visualizations of the placenta using a digital light microscope (the Nikon Eclipse Ci with Nikon DSR21 16 megapixels in 400 times magnification). Positive staining of immunohistochemistry is indicated in *golden brown color*. The intensity and percentage of cells stained representing an immunoreactive score convey placental expression of caspase-3, caspase-1, and TNF-alpha. *Yellow arrows* indicate the sites of positive staining on syncytiotrophoblast cells. *TNF-alpha*, tumor necrosis factor-alpha.

Bachnas. Placental damage comparison between Preeclampsia with COVID-19, COVID-19, and preeclampsia. Am J Obstet Gynecol Glob Rep 2023.

After IRS data collection, statistical analysis was performed to analyze the dependent variable (IRS) correlation with the independent variable (type of cases: preeclampsia, COVID-19, Preeclampsia with COVID-19) using IBM SPSS Statistics (version 26.0; IBM Corporation, Armonk, NY). Normality and homogeneity tests were performed using the Shapiro-Wilk and Levene tests. As the data were not normally distributed but homogenous, the statistical analysis was continued using the Kruskal-Wallis test. The quantitative data were presented as mean±standard deviation. A *P* value of <.05 was considered statistically significant.

## Results

A total of 72 pregnant women who were eligible and willing to participate were included in the study. There was no difference seen between the groups for the confounding factors (Table 1). We reported no maternal mortality during this study. Maternal age, parity, body mass index, and chronic disease were comparable between the 3 groups. Perinatal mortality was reported in 9 of 24 pregnancies complicated by both COVID-19 and preeclampsia (37.5%), 1 of 24 pregnancies complicated by preeclampsia (4.2%), and no case of pregnancies complicated by COVID-19. The 3 cases of intrauterine fetal death (IUFD) were reported in the Preeclampsia with COVID-19 group, and no case of IUFD was reported in the other 2 groups. Of note, 13 pregnant patients with both COVID-19 and preeclampsia (54.2%) were delivered before term, compared with 7 pregnant patients with preeclampsia only and COVID-19 only (29.2%). Statistical analysis on preterm birth comparison between groups was insignificant. Birthweights of <2500 g were found in 12 pregnancies complicated by both COVID-19 and preeclampsia (50.0%), 10 pregnancies complicated by preeclampsia (41.7%), and 5 pregnancies complicated by COVID-19 (20.8%). Asphyxia with an Apgar score of <7 was seen in 4 pregnancies complicated by both COVID-19 and preeclampsia (16.7%), 1 pregnancy complicated by COVID-19 (4.2%), and 4 pregnancies complicated by preeclampsia (16.7%). All pregnant patients with both COVID-19 and preeclampsia (100.0%), 15 pregnant patients with preeclampsia (62.5%), and 20 pregnant patients with COVID-19 (83.3%) were delivered abdominally.

**Table 1:** Characteristics of the participants between groups

Characteristics	Groups			*P* value
	Preeclampsia n (%)	COVID-19 n (%)	COVID-19 preeclampsia n (%)	
Maternal age (y)				.058
<35	21 (87.5)	21 (87.5)	15 (62.5)	
≥35	3 (12.5)	3 (12.5)	9 (37.5)	
Parity				.072
Primigravida	8 (33.3)	11 (45.8)	8 (33.3)	
Secundigravida and multigravida	12 (50.0)	6 (25.0)	0 (0)	
Body mass index				.858
Normal	2 (8.3)	2 (8.3)	7 (29.2)	
Overweight	19 (79.2)	21 (87.5)	11 (45.8)	
Obesity	3 (12.5)	1 (4.2)	6 (25.0)	
Chronic illness				.088
Yes	6 (25.0)	3 (12.5)	9 (37.5)	
No	18 (75.0)	21 (87.5)	15 (62.5)	
Mode of delivery				.003^a^
Vaginal	9 (37.5)	4 (16.7)	0 (0)	
Abdominal	15 (62.5)	20 (83.3)	24 (100.0)	
Gestational age at birth (wk)				.118
<37	7 (29.2)	7 (29.2)	13 (54.2)	
≥37	17 (70.8)	17 (70.8)	11 (45.8)	
Birthweight (g)				.099
<2500	10 (41.7)	5 (20.8)	12 (50.0)	
≥2500	14 (58.3)	19 (79.2)	12 (50.0)	
Apgar score				.319
<7	4 (16.7)	1 (4.2)	4 (16.7)	
>7	20 (83.3)	23 (95.8)	20 (83.3)	
Perinatal death				.002^a^
Survive	23 (95.8)	24 (100.0)	15 (62.5)	
IUFD	0 (0)	0 (0)	3 (12.5)	
Neonatal death (until 28 d)	1 (4.2)	0 (0)	6 (25.0)	

*IUFD*, intrauterine fetal death.

a A *P* value of <.05 is considered statistically significant.

Here, the apoptotic process was determined as the most prominent placental damage. This was well described from the caspase-3 placental expression, which showed an IRS of 11.92±2.15, a value that is near absolute (12). Concerning the other pathways, inflammation represented with caspase-1 placental expression and necrosis represented with TNF-alpha showed significant overexpression in all 3 groups, having average values above 4. Statistical significance was found between the 3 groups in all 3 pathways: caspase-3 (*P*=.044), caspase 1 (*P*=.031), and TNF-alpha (*P*=.024). Our data showed that the Preeclampsia with COVID-19 group had the poorest placental damage in all 3 pathways: apoptosis (11.92±2.15), inflammation (7.25±3.04), and necrosis (4.33±3.80). The COVID-19 group had the second poorest placenta damage (placental expression: caspase-3 [10.33±3.64], caspase-1 [5.71±4.29], and TNF-alpha [6.46±4.06]). The preeclampsia group had the least placental damage (placental expression: caspase-3 [9.88±3.25], caspase-1 [4.21±3.16], and TNF-alpha [7.42±4.28]). Although the result from the preeclampsia group was the least robust, when it is compared with the standard quantification in a normal placenta, it was still considered quite high. The results of the comparisons of caspase-3, caspase-1, and TNF-alpha placental expressions between the 3 groups can be seen in Table 2.

**Table 2:** Comparison of caspase-3, caspase-1, TNF-alpha placental expression between Preeclampsia with COVID-19, COVID-19, and preeclampsia

	Groups			
Immunoreactive score	Preeclampsia	COVID-19	Preeclampsia with COVID-19	*P* value
Caspase-3	9.88±3.25	10.33±3.64	11.92±2.15	.044^a^
Caspase-1	4.21±3.16	5.71±4.29	7.25±3.04	.031^a^
TNF-alpha	7.42±4.28	6.46±4.06	4.33±3.80	.024^a^

Data are presented as mean±standard deviation, unless otherwise indicated.

*TNF-alpha*, tumor necrosis factor-alpha.

a A *P* value of <.05 is considered statistically significant.

## Discussion

### Principal findings

This study showed that pregnancies complicated by both preeclampsia and COVID-19 result in perinatal death. Interestingly, these data were supported by the results of high expressions of caspase-3, caspase-1, and TNF-alpha in the placenta, which correlated with the destruction process of the placenta. The 3 major pathways of placental damage were apoptosis, inflammation, and necrosis. All 3 pathways were significantly higher in pregnancies complicated by both COVID-19 and preeclampsia. Surprisingly, pregnancies complicated by moderate to severe COVID-19 had more damaged placentas than pregnancies complicated by preeclampsia, although the interruption occurred over a shorter period. In addition, we can see that necrosis represented with TNF-alpha was more dominant than inflammation represented with caspase-1 in pregnancies complicated by preeclampsia only and pregnancies complicated by COVID-19 only; however, in pregnancies complicated by both COVID-19 and preeclampsia, this was vice-versa. This could explain the chronic inflammation that has already occurred because preeclampsia in conjunction with acute inflammation drives from COVID-19, causing higher inflammatory activity that can be potentially damaging to the placenta.

### Results in the context of what is known

From a previous study, placental apoptosis has been found to be increased in preeclampsia, and this is associated with activation of caspase-3.^24^ This is in line with the result of this study. In COVID-19, there was no proper publication about the placental expression of caspase-3, but some publications about placental pathology have shown significant events of apoptosis.^25^ If we compare any mean of the study, our result is in line with the previous research by Hajizadeh Maleki and Tartibian,^26^ which showed that caspase-3 serum levels of patients with COVID-19 were higher than normal and correlated with the severity levels of COVID-19.^26^^,^^27^ Until this research was completed, there was no publication that explained the pathology of the placenta in pregnancies complicated by both COVID-19 and preeclampsia. Moreover, this addressed caspase-3, caspase-1, and TNF-alpha expressions. Nevertheless, some believe that there are published works related to preeclampsia and COVID-19 and there conjunction with the pathophysiological process.^4^^,^^5^^,^^14^^,^^28^ Emerging results on increasing production of caspase serum in patients with COVID-19 showed robust results, including caspase-1.^12^ In our study, placental expression of caspase-1 from patients with COVID-19 was high and was even higher if it was accompanied with preeclampsia. This could not be compared with any other publications as such publications do not exist yet. In contrast, when this is compared with the publication on preeclampsia only, we can see matching results with significantly increased expressions.^29^^,^^30^ The severity of necrosis in the placenta has been shown in both preeclampsia and COVID-19, but there is no solid publication to explain how it is in Preeclampsia with COVID-19.^9^^,^^25^ This recent study has shown that Preeclampsia with COVID-19 had the worst necrosis condition in the placenta compared with COVID-19 only or preeclampsia only, which is demonstrated by TNF-alpha expression.

### Clinical implications

Here, we can learn about the biomechanism of placental damage in Preeclampsia with COVID-19, COVID-19, and preeclampsia at least from the 3 aspects, which are apoptotic process, inflammation, and necrosis. In addition, we can generate new ideas with this standpoint on how to manage patients with both COVID-19 and preeclampsia. Giving medications, such as antiapoptotic or anti-inflammatory agents, and keeping sufficient oxygenation to the placenta will prevent placental damage and result in better perinatal outcomes.

### Research implications

In the future, clinical trials are needed to support the idea of “placenta protection management” in Preeclampsia with COVID-19 cases so that poor perinatal outcomes could be prevented.

### Strengths and limitations

This study could give an insight into how Preeclampsia with COVID-19 could be very damaging to the placenta, resulting in poor perinatal outcomes. Nevertheless, there are limitations to this study, such as the various treatment strategies in severe cases of COVID-19, which could be better adjusted in the upcoming research and give more punctual quantification and analysis.

### Conclusions

Placental expressions of caspase-3, caspase-1, and TNF-alpha were significantly high in pregnancies complicated by both COVID-19 and preeclampsia, suggesting highly morbid placental damage with unfavorable perinatal outcomes.
